# A Model Procedure for Catalytic Conversion of Waste Cotton into Useful Chemicals

**DOI:** 10.3390/ma14081981

**Published:** 2021-04-15

**Authors:** Michal J. Binczarski, Justyna Malinowska, Andrei Stanishevsky, Courtney J. Severino, Riley Yager, Malgorzata Cieslak, Izabela A. Witonska

**Affiliations:** 1 Institute of General and Ecological Chemistry, Lodz University of Technology, 116 Zeromskiego Street, 90-924 Lodz, Poland; michal.binczarski@p.lodz.pl (M.J.B.); justyna.malinowska@dokt.p.lodz.pl (J.M.); 2 Department of Physics, University of Alabama at Birmingham, 1300 University Blvd., Birmingham, AL 35294, USA; astan@uab.edu (A.S.); cosev@uab.edu (C.J.S.); ryager7@uab.edu (R.Y.); 3 ŁUKASIEWICZ Research Network—Textile Institute, Department of Textile Chemical Technologies, 118 Gdanska Street, 90-520 Lodz, Poland; cieslakm@iw.lodz.pl

**Keywords:** cotton waste utilization, acidic hydrolysis, Pd-Au/SiO_2_ catalyst

## Abstract

Cotton is grown in about 90 countries and accounts for 24% of the fibers used in the global production of textiles. In 2018/2019, 25.8 Mt of cotton were produced around the world. Since this natural product consists mainly of cellulose, it can be used as a raw material in the so-called “sugar economy”. This paper discusses a model procedure for thermally assisted acidic hydrolysis of cotton into glucose and subsequent oxidation of the glucose into calcium gluconate over Pd-Au/SiO_2_ catalyst. In the first step, H_2_SO_4_ was used as a catalyst for hydrolysis. The cotton hydrolysates were neutralized using CaCO_3_ and applied as a substrate in the second step, where glucose was oxidized over Pd-Au/SiO_2_ prepared by ultrasound assisted co-impregnation. With the appropriate Au/Pd molar ratio, small crystallites of palladium and gold were created which were active and selective towards the formation of gluconate ions. This approach to the transformation of glucose represents as a viable alternative to biological processes using fungal and bacterial species, which are sensitive to the presence of inhibitors such as furfurals and levulinic acid in hydrolysates.

## 1. Introduction

The global demand for textiles is growing steadily, and looks set to continue rising [1,2,3]. In 2013, the global production of textile materials was around 85.5 Mt, and by 2025 it is estimated to increase to 130 Mt [4]. Textiles are used in a vast range of products, including clothing, bed linen, towels, and utility fabrics, which quickly become worn-out or fall out of fashion, ending up in landfills. As much as 63% of the textile fibers currently sold are derived from petrochemicals [5] and only 37% from natural fibers—mainly cotton (24%) [6], wool or linen. As well as direct and reactive dyes, pigments, components of printing pastes, polyacrylic coatings, and polyurethane coatings, many chemical modifiers are used to impart cotton textiles with special functions [7,8]. These chemicals include hydrophobic, oleophobic, and antistatic finishing agents, flame retardants, and UV radiation absorbers. Many such chemicals prevent the natural biodegradation of textiles, in part since they inhibit the growth of microorganisms (fungi, bacteria, etc.) [9]. Therefore, textiles constitute a significant group of wastes, which are difficult to biodegrade in the natural environment. Many low-quality cotton products are unsuitable for material recycling and reuse.

A possible solution is to convert cotton textiles by chemical-biological methods into useful energy products, such as biogas or into simple sugars, such as glucose. Via chemical or biological transformations, these products can then be made into valuable chemicals such as gluconic acid [10,11,12,13] or into other products such as hydrogen, vanillin or monolayer-patched graphene [14,15,16,17]. The catalytic transformation of glucose into gluconic acid over heterogeneous catalysts is of great interest to the industry [18,19,20,21,22,23]. Despite considerable economic and environmental interest in the development of methods for processing waste textiles for use as fermentation media or chemical substrates, there has been little discussion of this topic in the literature [24,25,26,27]. Preliminary studies show that even fabrics based on natural fibers (cotton, wool) only but subjected to a chemical treatment are not easily biodegradable in the natural environment, due to the presence of fermentation inhibitors such as furfurals and levulinic acid [28]. Therefore, catalytic transformation seems a better solution than biological methods to the problem of textile waste. 

**Figure 1:**
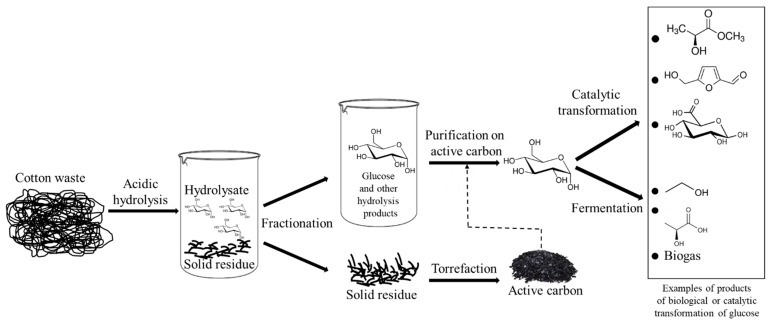
Scheme of cotton waste management, including acid hydrolysis and further chemical and biological processes to convert glucose into useful chemical products.

Figure 1 shows a process for treating cotton waste, including textiles with synthetic additives (polyester and polyamide). It is based on acid hydrolysis of cellulose and further biological or catalytic transformation of the glucose into useful chemical products. Only some of the products that can be obtained from glucose are indicated in the diagram. A very important product for ensuring a waste-free process is activated carbon, which can be made from the solid residues after hydrolysis of cotton waste. The activated carbon may be used in the process itself to purify the hydrolysate from fermentation inhibitors or sold as a commercial product. Research efforts at Lodz University of Technology to torrefy the solid residue of cotton fibers after hydrolysis have shown that activated carbon with a high specific surface area (>500 m^2^/g) can be produced, which is capable of adsorbing the non-sugar components of hydrolysates. Lactic bacteria and yeast were successfully multiplied on hydrolysates purified on a bed of activated carbon produced from cotton waste. This research is ongoing and will be the subject of forthcoming publications.

In this paper, a model procedure for thermally assisted acidic hydrolysis of cotton materials is presented and discussed. Hydrolysates obtained from raw cotton yarn were used as the substrate for the catalytic transformation of glucose into gluconic acid over Pd-Au/SiO_2_ catalysts. Bimetallic palladium–gold systems were prepared at Lodz University of Technology and used initially for the hydrogenation of furfural [29]. When it was observed that the bimetallic systems effectively catalyzed hydrogen transfer reactions, they were used for the oxidation of glucose, which occurs according to the oxidative dehydrogenation mechanism.

## 2. Materials and Methods

### 2.1. Catalyst Preparation

The PdCl_2_ solution (POCH S.A., Gliwice, Poland, pure for analysis) acidified to about pH 5 (HCl_aq_, 35–38%, pure for analysis) (CHEMPUR, Piekary Slaskie, Poland) was used to prepare a 5% Pd/SiO_2_ catalyst by wetness impregnation of SiO_2_ (Sigma-Aldrich Chemie GmbH, Taufkirchen, Germany, 291 m^2^/g). The preparation was left in an ultrasonic bath with a frequency of 45 kHz for 24 h. The water was evaporated by heating to 60 °C under a vacuum. The catalyst was air dried at 110 °C for 6 h then reduced for 2 h at 300 °C under an H_2_ atmosphere (Air Products, Warsaw, Poland, Premium Plus, 99.999%, 20 cm^3^·min^−1^). A linear temperature increase of 20 °C·min^−1^ was set for all stages of thermal processing. The same procedure was applied to prepare a 5% Au/SiO_2_ (wt%) monometallic catalyst from an AuCl_3_ solution (POCH S.A., Gliwice, Poland, 0.21 wt% Au). 

Pd-Au/SiO_2_ catalysts containing 5 wt% Pd and 0.2–10 wt% Au were prepared by co-impregnation of a SiO_2_ carrier with solutions of AuCl_3_ (POCH, pure, 0.21% Au) and PdCl_2_ (POCH, anhydrous, pure for analysis), according to the same procedure. All systems were subjected to ICP-AES (IRIS AP, Thermo Jarrel Ash, Waltham, MA, USA), XRD (PANalytical, Malvern, UK), ToF-SIMS (ToF-SIMS IV, ION-TOF GmbH, Münster, Germany), SEM-EDS (SEM S-4700, Hitachi, Tokyo, Japan; EDS, Thermo-Noran Inc., Madison, WI, USA), and XPS (VersaProbe 5000, PHI, Chanhassen, MN, USA) analysis to determine their compositions and identify metal interactions which could affect their catalytic properties in the glucose oxidation process. 

### 2.2. Catalyst Measurements

A glucose solution (GLU, POCH S.A. Gliwice, Poland, pure for analysis; C_0 GLU_ = 1.00 mol·dm^−3^; V_GLU_ = 0.25·dm^3^) was oxidized in a 0.4 dm^3^ thermostated reactor (Parr Company, Moline, IL, USA) equipped with an O_2_ supply system (Air Products, Pure N 5.2 UP, 99.9992%), a stirrer, a pH electrode (Mettler-Toledo Sp. z o.o., Warsaw, Poland), and a burette containing NaOH (POCh Gliwice, Poland, pure p.a.; C_NaOH_ 0.50 mol·dm^−3^). The reaction was performed at pH 9, 60 °C. In each reaction run 1 g of catalyst was used. While the mixture was being stirred at 1300 rpm, O_2_ was bubbled through at 0.9 dm^3^·min^−1^. Samples of the reaction medium were taken periodically for analysis. The samples were filtered and analyzed using a LaChrom liquid chromatograph (Marck-Hitachi, Tokyo, Japan) coupled to a UV–Vis detector (Marck-Hitachi, Tokyo, Japan), with an analytical wavelength of 200 nm. The reaction products were separated on a 150 × 3.3 mm ID amino-propylo-silicone column. A water solution of acetonitrile (70% ACN, 30% phosphate buffer pH 5.5) was used as a mobile phase. 

A Gilson’s prepELS II detector (Gilson Company, Inc. Lewis Center, OH, USA) was used as an additional analyzer in the HPLC system (Marck-Hitachi, Tokyo, Japan). The temperature of the spray chamber was 15 °C. The temperature of the drift tube was 85 °C. With these parameters, the retention times of all the considered compounds were: t_fructose_ = 0.988 min; t_glucose_ = 1.425 min; t_gluconic acid_ = 8.230 min; t_2-keto-gluconic acid_ = 13.743 min; t_guluronic acid_ = 15.260 min; t_glucaric acid_ = 17.250 min, and it was possible to identify and determine the amounts of these compounds.

Catalytic results were expressed as the conversion degree (X, %) and selectivity (S, %), defined as:

X (%) = (1 − (C_GLU_/C_0 GLU_)) × 100%

S (%) = (C_PROD_/(C_0 GLU_ − C_GLU_)) × 100%

where C_0 GLU_ is the molar concentration of glucose at the beginning of the oxidation process, C_GLU_ is the molar concentration of glucose over time, and C_PROD_ is the molar concentration of the product (fructose, gluconic acid, 2-keto-gluconic acid, guluronic acid, glucaric acid) over time. 

### 2.3. Synthesis of Glucose from Cotton Yarn

A water solution of glucose was obtained by acidic hydrolysis (H_2_SO_4_, analytical grade, 95%, Stanlab, Lublin, Poland) of cotton yarn Z689 × 2 S 542 (linear density 58.73 ± 0.641 tex, fiber length 15–32 mm, specific strength 18.44 ± 1.089 cN/tex). Cotton yarn hydrolysis was performed in a pressure reactor (Parr Instrument Company Series Mini 4552) with a volume of 7.5 dm^3^ at a temperature of 120–160 °C. The start of the hydrolysis process was considered to be the moment when the reaction mixture reached the desired temperature. In each run, 100 g of comminuted cotton yarn (length > 0.5 cm) and 2.0 dm^3^ of a solution of H_2_SO_4_ (concentration 0.5–10%) were used. After 1–4 h, the reaction mixture was cooled in the reactor to room temperature and made alkaline by adding CaCO_3_ (analytical grade, POCh, Gliwice, Poland) to pH 7.5. The mixture was then filtered through a bed of activated carbon and the glucose concentration was measured by high performance liquid chromatography (HPLC) using a SYKAM HPLC system with a S1125 HPLC Pump System, S 5300 autosampler, S 4115 column thermostat, and S 3585 RI detector (Sykam GmbH, Eresing, Germany). The sugars were separated on a SETREX IEX-H^+^ column (300 × 8.0 mm ID) at 80 °C, using 0.008 mol·dm^−3^ H_2_SO_4_ + 2%(v/v%) ACN (flow 0.8 cm^3^ min^−1^) as a mobile phase. The quantitative analysis of glucose was performed based on the calibration curve for the range of glucose concentrations at 0–10 g dm^−3^ (the curve in the analyzed range was linear y = 0.19733 x, R^2^ = 0.9998803).

### 2.4. Analytical Techniques Used for Measurements of Bimetallic Pd-Au/SiO_2_ Catalysts

A PANalytical X’Pert Pro MPD diffractometer (PANalytical, Malvern, UK) equipped with a PANalytical X’Celerator detector (PANalytical) was used to acquire X-ray diffraction patterns. The operating parameters for the Cu X-ray tube source were 40 kV voltage and 30 mA current. The XRD patterns were recorded from 20 to 90° 2θ with a 0.0167° step value and a 20 s dwell time. The crystalline phase analysis was performed using the ICDD PDF−2 (version 2004) database.

The temperature programmed reduction (TPR) was performed using a flow apparatus [30]. Prior to the analysis, 0.1 g samples of the oxidized catalyst were flushed with argon at room temperature for 0.5 h. The TPR measurements were then performed at 25–500 °C with a linear temperature increase of 20 °C·min^−1^ under 5 vol% of H_2_ in Ar at a flow rate of 30 cm^3^·min^−1^. 

The secondary ion mass spectra of the bimetallic catalyst samples (formed into tablets) were recorded using a secondary ion mass spectrometer (ToF-SIMS IV, ION-TOF GmbH, Münster, Germany) coupled to a ToF-SIMS IV time of flight detector (ToF-SIMS IV, ION-TOF GmbH, Münster, Germany, liquid metal ^69^Ga^+^ ion gun). An area on the sample surface 100 × 100 µm^2^ in size was irradiated with 25 keV pulses of ^69^Ga^+^ ions at a 10 kHz repetition rate. The average ion current was 2.5 pA. As a result of the short analysis time (50 s), the ion dose was below the static limit of 1 × 10^13^ ions/cm^2^. High resolution images were recorded using either the extreme crossover mode or the burst alignment mode. The ToF-SIMS spectra of the catalyst samples were recorded with a primary ion pulse width of 650 ns, a high mass resolution (m/Δm), and 29 mass units (m.s.) normally greater than 8000. 

A scanning electron microscope (SEM S-4700, Hitachi, Tokyo, Japan) equipped with an energy dispersive spectrometer (EDS, Thermo-Noran Inc., Madison, WI, USA) was used for the SEM analysis of the catalysts. Samples were embedded in conductive carbon pads and the excess loose powder was removed. To reduce electric charging, the samples were sputter coated with carbon (Cressington 208 HR system). Images were acquired in a back-scattered electron (BSE) mode. An accelerating voltage of 25 kV was used. For the purposes of comparison, the same samples were analyzed using an FEI Quanta 650 SEM (FEI Company, Hillsboro, OR, USA) equipped with a Bruker Energy Dispersive Spectroscopy (EDS) system (Bruker Corporation, Billerica, MA, USA). A 15 kV accelerating voltage, 3.5 µA electron beam current, and 10 mm working distance were used. The compositions of each sample were measured at least three times at different locations approximately 0.25 mm^2^ in size. 

Samples were prepared by pressing the catalytic powders into high-purity carbon filled conductive tabs (PELCO Tabs™). An X-ray photoelectron spectroscope (XPS, PHI VersaProbe 5000, PHI, Chanhassen, MN, USA) was used to analyze the chemical bonding on the surface of the Pd-Au/SiO_2_ catalysts. A focused monochromatic Al K-α source (E = 1486.6 eV; 100 µm spot diameter) was used to record survey and high-resolution spectra. Induced electric charge compensation was measured using a cold cathode electron flood gun and a low-energy Ar^+^ ion-beam source. To avoid ion-induced surface damage, ion sputtering was not used. The recorded XPS spectra were fitted using a Multipak software package. The spectra were compared both to the NIST database (NIST Standard Reference Database 20, version 3.5) and to previously published data [29].

### 2.5. Studies of Catalyst Stability in the Reaction Mixture

Gold and palladium losses from the catalysts in the reaction mixture were determined by analyzing the filtered solution by inductively coupled plasma-atomic emission spectroscopy (ICP-AES), using an IRIS AP optical emission spectrometer (IRIS AP, Thermo Jarrel Ash, Waltham, MA, USA) with horizontal observation of the plasma. A MLS–1200 Mega Microwave Digestion System (Mile-stone) was used for complete digestion of the samples prior to the ICP-AES analysis.

## 3. Results

In the previous research, we examined the catalytic properties of monometallic Pd/SiO_2_ catalysts (0.5–10% by weight of Pd). It was observed that the amount of gluconic acid produced in the reaction increased with the percentage content of palladium. The highest selectivity was shown by the samples containing 5–10 wt% Pd/SiO_2_. These catalysts had practically the same selectivity. Therefore, adding more than 5 wt% of palladium into catalytic systems appears to be of no advantage from either an economic or practical point of view. Commercial catalysts recommended by manufacturers for use in liquid phase oxidation processes (e.g., Degussa catalyst) also contain about 5 wt% active metal, which means that the catalytic properties of the catalysts obtained in this study are comparable with commercial catalysts.

**Figure 2:**
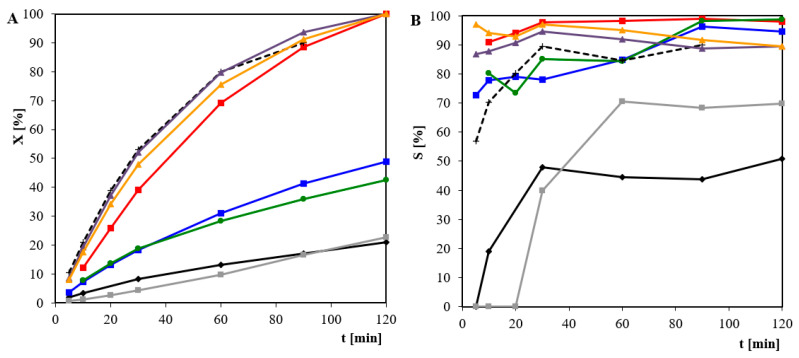
(**A**) Conversion degree of glucose X [%]; (**B**) selectivity to gluconate ions S_GLC_ [%] of different catalysts: 5% Pd/SiO_2_ (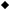); 5% Au/SiO_2_ (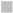); 5% Pd-0.2% Au/SiO_2_ (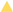); 5% Pd-0.5% Au/SiO_2_ (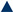); 5% Pd-1% Au/SiO_2_ (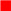); 5% Pd-2% Au/SiO_2_ (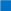); 5% Pd-5% Au/SiO_2_ (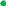), and 1% Pt-4% Pd-5% Bi/C (Degussa) (×) as a function of time. Reaction conditions: T = 60 °C, m_cat_ = 1g, C_0_ = 1 mol·dm^−3^, pH = 9, V_O_2__ = 1 dm^3^·min^−1^.

Figure 2 shows the dependence of conversion and selectivity towards gluconic ions on the time of glucose oxidation over palladium-gold (Pd-Au) catalysts on silica. A 1 mol·dm^−3^ solution of pure commercial glucose in water was used as a substrate. Monometallic catalysts (5% Pd/SiO_2_ and 5% Au/SiO_2_) showed rather poor activity. In general, the addition of gold to the 5% Pd/SiO_2_ systems caused an increase in the conversion degree of glucose. The addition of only a small amount of gold to the palladium catalysts (0.2–1 wt%) had a significant effect on the activity of the catalyst. However, in the case of 5% Pd-2% Au/SiO_2_ and 5% Pd-5% Au/SiO_2_ compared to monometallic catalysts, the increase in activity was lower than that in the bimetallic systems with lower amounts of gold. Therefore, amounts of gold higher than 1 wt% are counterproductive in Pd-Au/SiO_2_ catalysts.

All the studied bimetallic catalysts were characterized by high selectivity towards gluconic ions. In all cases, the selectivity of the Pd-Au/SiO_2_ systems was comparable or even higher than in the case of the commercial Degussa catalyst (1% Pt-4% Pd-5% Bi/C). 

To understand the role of the Pd-Au/SiO_2_ systems in the oxidation of glucose into gluconic acid in aqueous phase, the catalysts were analyzed using scanning electron microscopy (SEM), time-of-flight secondary ion mass spectroscopy (ToF-SIMS), X-ray diffraction (XRD), energy-dispersive x-ray spectroscopy (SEM-EDS), x-ray photoelectron spectroscopy (XPS), and temperature programmed reduction (TPR). Detailed results of these physicochemical tests are given in [29].

**Figure 3:**
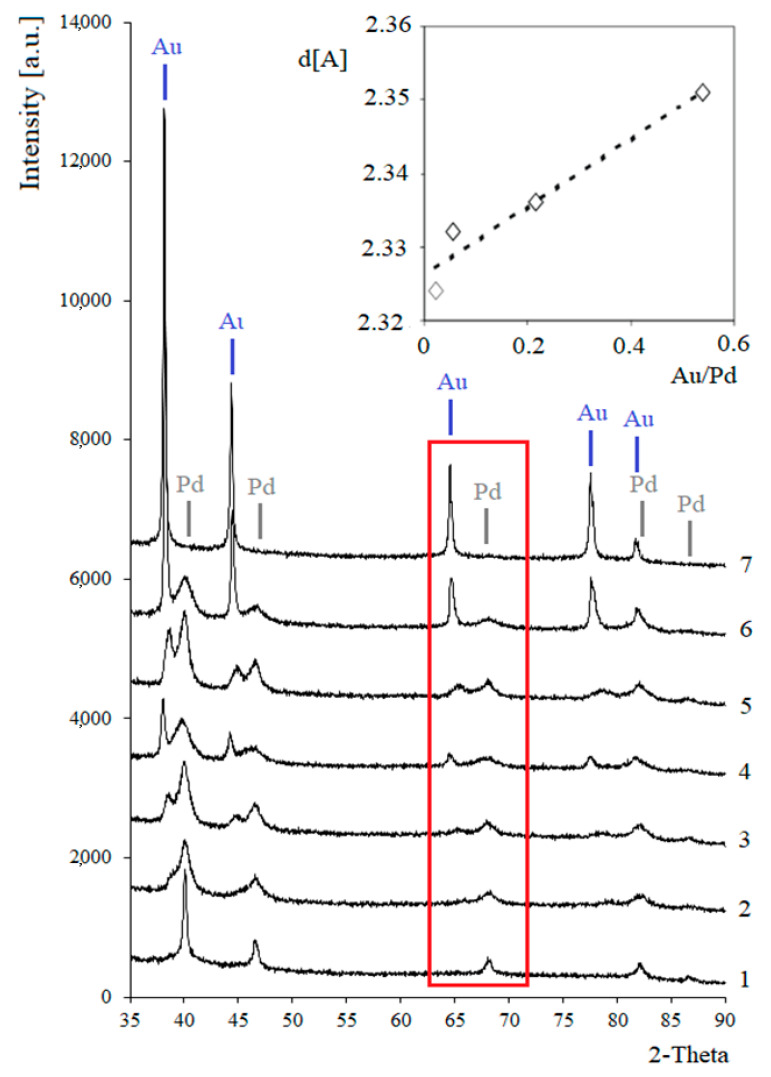
X-ray diffraction patterns for Pd-Au/SiO_2_ catalysts: (1) 5% Pd/SiO_2_; (2) 5% Pd-0.2% Au/SiO_2_; (3) 5% Pd-0.5% Au/SiO_2_; (4) 5% Pd-1% Au/SiO_2_; (5) 5% Pd-2% Au/SiO_2_; (6) 5% Pd-5% Au/SiO_2_; (7) 5% Au/SiO_2_ and the linear relationship of interplanar distances in the composition function of these systems (Vegard’s rule). The bimetallic catalysts were activated in a hydrogen atmosphere for 2 h at 300 °C. Adapted from ref. [29].

The XRD patterns show the maxima assigned to the metallic Pd phase (Figure 3). The introduction of gold into the monometallic system was associated with the appearance of additional maxima on the diffraction patterns. The positions of the maxima shifted towards smaller values of the angle 2θ. The positions of the maxima in the diffractogram of the 5% Pd-5% Au/SiO_2_ catalyst are close to the maxima corresponding to the gold phase. These results indicate the possible formation of solid solutions. In the case of the Pd-Au/SiO_2_ systems activated in a hydrogen atmosphere at 300 °C, confirmation of the formation of solid solutions is given by the linear relationship between the interplanar distances as a function of the alloy composition, according to Vegard’s rule. No additional diffraction maxima were found in the diffraction patterns of the bimetallic systems, but only the maxima for the metallic Pd and Au phases. This indicates a lack of strong interactions between these metals, which would be manifested by the formation of intermetallic compounds. The results of the XRD tests were confirmed by XPS and TRR tests [29].

The addition of a larger amount of gold (>2 wt%) led to an increase in the intensity of the sharper diffraction peaks attributed to gold metal, which is evidence of the existence of larger Au crystallites on the surface of the bimetallic catalysts. Using the Scherrer equation, the average sizes of the palladium and gold crystallites were estimated. The results shown in Table 1 reveal that the palladium dispersion was greater in the case of bimetallic systems than for the monometallic 5% Pd/SiO_2_ catalyst. Better Pd dispersion in the bimetallic systems may explain their higher activity and selectivity for gluconic acid. Moreover, the smallest Au crystallites formed on the 5% Pd-1% Au/SiO_2_ catalyst. It is well known that the catalytic properties of gold strongly depend on the size of the gold particles [31]. Data in the literature show that monometallic gold carrier systems are catalytically active in the process of glucose oxidation when the Au particle size is less than 10 nm [32]. In the case of the 5% Pd-1% Au/SiO_2_ catalyst, the estimated Au particle size was 8 nm. This catalyst also showed good catalytic properties for the oxidation of glucose to gluconic acid. Therefore, it cannot be ruled out that gold contributed to the increase in catalytic activity.

Images obtained by ToF-SIMS of the Pd-Au/SiO_2_ catalysts showed a nonhomogeneous distribution of Au on the silica surface [29]. The bimetallic catalysts tended to form Au-enriched regions, which increased in size as more gold was introduced into the system. The distribution of the Pd fraction was better in the case of the bimetallic systems than in that of the monometallic catalyst 5% Pd/SiO_2_. Moreover, the calculated relative intensities of the Au_3_/Pd peaks revealed that the number of surface gold atoms was highest in the case of the 5% Pd-1% Au/SiO_2_ system, which also showed the highest activity in the glucose oxidation reaction. This result also indicates good dispersion of Pd. On the other hand, the comparable intensities of the Pd peaks obtained for the bimetallic Pd-Au catalysts suggest that these systems had similar numbers of surface palladium atoms (Table 2). It can be concluded that in the case of all the tested Pd-Au/SiO_2_ catalysts the Pd atoms were similarly distributed on the support surfaces. These results of ToF-SIMS tests are in good agreement with those obtained with the XRD technique.

**Figure 4:**
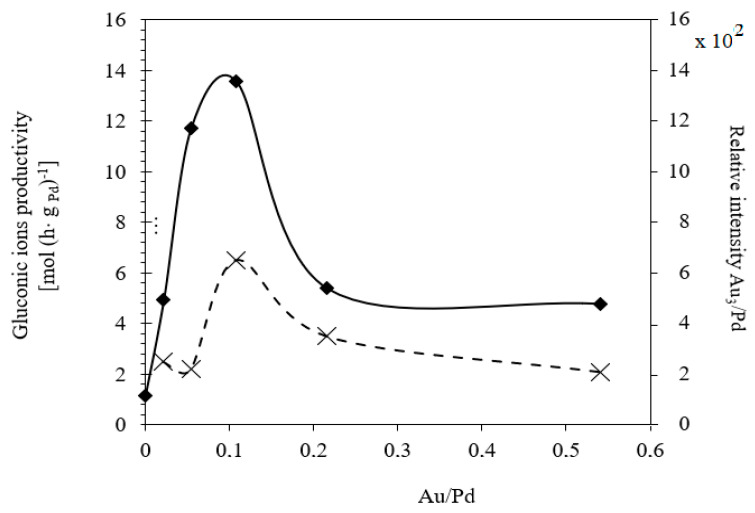
Gluconic ions productivity and relative intensity of Au_3_/Pd ions as a function of the atomic ratio of Au/Pd in bimetallic Pd-Au/SiO_2_ catalysts. Productivity was estimated after 2 h runs over bimetallic catalysts with various compositions of the active phase. Reaction conditions: T = 60 °C, m_cat_ = 1g, C_0_ = 1 mol·dm^−3^, pH = 9, V_O2_ = 1 dm^3^·min^−1^.

Figure 4 shows the dependence of the relative intensities of Au_3_/Pd as a function of the Au/Pd atomic ratio in bimetallic catalysts. As can be seen, the largest number of Au atoms was measured on the catalyst surface of the 5% Pd-1% Au/SiO_2_ system. This catalyst also showed the highest rate of gluconic acid formation. Thus, it can be assumed that the presence of both metals on the support may be responsible for the activity of the bimetallic system. Confirmation of good gold dispersion on the 5% Pd-1% Au/SiO_2_ bimetallic system was also provided by previously conducted microscopic examinations (SEM-EDX) and CO chemisorption measurements [29].

Gluconate ions are good chelates for metal ions. Therefore, we studied the degree of leaching of Pd and Au ions from the catalysts during the glucose oxidation process. The results of inductively coupled plasma-atomic emission spectroscopy (ICP-AES) test performed on real solutions after glucose oxidation processes are presented in Table 3. In the case of the Pd-Au/SiO_2_ catalysts, leaching of Pd only was observed. The amount of Pd lost was comparable for all bimetallic catalysts. Gold was not detected in the reaction mixture when the process was conducted over bimetallic Pd-Au/SiO_2_ catalysts. However, in the case of monometallic 5% Au/SiO_2_ catalysts the presence of a trace amount of gold was confirmed by ICP-AES measurements.

The application of catalysts in technological processes is economically feasible and justifiable only when they show long-term stability. For this reason, the stability of the 5% Pd-1% Au/SiO_2_ catalyst, which provided the best gluconic acid productivity during the reaction of glucose oxidation in liquid phase, was investigated (Table 4). The stability of the catalyst was estimated based on activity changes during 10 measurement cycles lasting 2 h each. The process of oxidation was conducted for a steady amount of catalyst (m_cat_ = 1 g), which was not removed from the reaction mixture in the subsequent cycles. After each measurement cycle, the reaction mixture was decanted and examined by HPLC and ICP-AES. A fresh charge of glucose solution (V = 0.25 dm^3^, C_0_ = 1 mol·dm^−3^) was then introduced into the reactor. The bimetallic 5% Pd-1% Au/SiO_2_ system was characterized by high stability in the reaction. After 10 reaction cycles, the activity and selectivity of this catalyst were practically the same as their original values. Additionally, ICP-AES measurements of the reaction mixture executed after each cycle proved the good stability of the bimetallic systems under the reaction conditions. Only a trace amount of palladium was detected in the reaction mixture after each cycle (Table 3). This high stability of the 5% Pd-1% Au/SiO_2_ system in the oxidation of glucose is an advantage for industrial applications. 

Cotton fibers consist almost entirely of cellulose (94–100%) and cotton hydrolysis therefore leads to glucose as a main product. Acidic hydrolysis of the cotton yarn was performed in a pressure reactor. Prior to HPLC analysis, samples of the hydrolysates were neutralized with milk of lime to pH 7.5, filtered through a bed of activated carbon (ERCARBON GE, 100 g), and deposited on a vacuum filter. Based on the data collected in Table 5, it can be seen that an increase in the hydrolysis temperature from 80 to 160 °C, together with an increase in the process time from 1 to 4 h, favors the depolymerization of cellulose and the formation of larger amounts of glucose. However, analysis of the mass spectra using GC-MS techniques revealed the presence of (4.970) furfural and (8.558) 2-furanocarboxaldehyde, 5-methyl- in the sample of the hydrolysate obtained at 120 °C. Increasing the temperature up to 160 °C led to the creation of products in addition to furfural and 2-furanocarboxaldehyde, 5-methyl-: (7.885) 2(5H)-furanone, 5-methyl- and (12.438) levulinic acid. On the other hand, raising the concentration of sulfuric acid (VI), which catalyzes the hydrolysis of polysaccharides, to more than 5% did not cause further increases in the concentration of sugar in the hydrolysates. The SEM images of the solid residue after hydrolysis of the cotton fibers clearly show that the most significant change in the morphology of the fibers occurred when the process was carried out at a high temperature (160 °C). The highest concentration of glucose was also noted in this sample (0.02 mol/dm^3^). For this reason, the hydrolyzate obtained by the reaction of 100 g of cotton fiber with 2 dm^3^ of 2% H_2_SO_4_ at a temperature of 160 °C for 2 h was selected for further catalytic tests.

The degree of crystallinity of cellulose was analyzed for cotton fibers and for the solid cotton residues after hydrolysis. A simplified method (Segal peak height method) was used to estimate the crystallinity of cellulose. The results tended to overestimate the degree of crystallinity, although the relative changes in the values for crystallinity enabled a comparative analysis of the results, which are summarized in Table 5. Increases in the degree of crystallinity of cellulose indicate more effective depolymerization of amorphous cellulose in the samples.

The hydrolysate obtained from the reaction of 100 g of cotton fiber with 2 dm^3^ of 2% H_2_SO_4_ at 160 °C for 2 h was used as a substrate in catalytic tests. The results of glucose oxidation are presented in Table 6. Catalytic reactions were performed over 5% Pd-1% Au/SiO_2_ catalyst with three concentrations of glucose (the hydrolysate was concentrated two and four times by evaporating the solvent at 60 °C). The catalyst was used in amounts corresponding to the catalyst loading in glucose solutions of 1 mol·dm^−3^ (Table 1, m_cat_ = 1 g, C_0 GLU_ = 1 mol·dm^−3^, V _GLU_ = 0.25 dm^3^).

The results confirmed the possibility of chemically processing cotton waste into sodium gluconate.

## 4. Discussion

The addition of a second metal (Bi, Tl, Sn, Pb, Ag) to supported palladium systems is a recognized way of modifying their catalytic properties, especially selectivity, for the oxidation of aldoses (glucose, lactose) to aldonic acids (gluconic acid, lactobionic acid) [19,20,21,22,23]. However, the principles behind such modifications are still a matter of discussion in the literature [19,20,21,22,23]. Some authors have tried to define the mechanism by which gold acts as a metallic promoter of palladium-based supported catalysts [33,34,35,36]. Enache et al. [33] suggested that Au acts as an electronic promoter for Pd in the oxidation of primary alcohols to aldehydes, so the electronic effect is dominant. On the other hand, Roudgar et al. [34] argued that geometric effects were more important than electronic effects, when they used Pd-Au film to promote the synthesis of vinyl acetate. In turn, Baddeley et al. [35] and Gleich et al. [36] suggested that the formation of surface ensembles of Pd_x_Au_y_ plays an essential role in improving reactivity.

Previous studies have indicated that glucose oxidation is sensitive to the structure of the bi-metallic catalyst [18,19,20,21,22,23]. For this reason, the ratio of Pd atoms to Au atoms in the Pd-Au/SiO_2_ bimetallic catalyst can significantly impact its catalytic properties. Among the studied catalysts with Au/Pd atomic ratios in the range of 0.0–0.55 [29], the system with an Au/Pd atomic ratio of about 0.1 (determined for the 5% Pd-1% Au/SiO2 catalyst) showed the best activity and the highest selectivity to gluconic acid. On the basis of XRD, ToF-SIMS, and previous SEM-EDS and XPS [29] studies, it was concluded that in this system these metals had the highest dispersion on the support and were also in direct contact, which explains the high selectivity of this catalyst. The addition of gold to Pd/SiO_2_ catalysts led to the stabilization of small Pd particles, and to the formation a Pd-Au alloy (the composition of which strongly depended on the Au/Pd atomic ratio). Despite the fact that gluconic acid has been shown to strongly adsorb on the surface of palladium, the bimetallic 5% Pd–1% Au/SiO_2_ catalyst was stable under the reaction conditions. Even after 10 reaction cycles, we observed high activity and selectivity towards gluconic acid. Thus, the addition of gold can prevent Pd poisoning, as well as improve the activity, selectivity, and stability of supported palladium systems.

The research presented in this publication is a part of a larger project, which includes the development of a procedure for temperature-assisted acid hydrolysis of cotton waste and further conversion of the obtained sugars into chemical and biotechnological products. In future work, we will present a concept for acidic hydrolysis of cotton waste to sugars, which can be converted via biological processes into fuels, especially biogas rich in hydrogen. Other studies in the literature have demonstrated that cotton waste can be used for the production of ethanol, but most often the first stage is enzymatic hydrolysis of the raw material [37,38,39]. This type of hydrolysis may be effective, but it is expensive due to the high cost of enzymes. For this reason, attempts have been made to employ the cheaper method of acidic hydrolysis. Various hydrolysis strategies are available, but some of the most widely used involve treatment with either concentrated or diluted acids. Diluted acids require high temperatures [40,41,42,43]. Since methods for acid hydrolysis of textile waste have not been implemented commercially and the results published in the literature most often concern only acidic hydrolysis of pure cellulose, rather than cotton or textile wastes, it is necessary to perform basic laboratory tests to establish the conditions for acidic hydrolysis of well-defined textile samples and the compositions of the hydrolysates. The biological investigations currently underway will enable a precise characterization of the fermentation inhibitors present in the fabric hydrolysates. This will enable the composition and finishing of fabrics to be correlated to the composition of the hydrolysates. A model will then be elaborated for treating textile waste so it can be used as a medium in biogas installations. 

## 5. Conclusions

The transformation of organic waste into valuable chemical substances has attracted much interest, due to its great practical and commercial potential. One such transformation is the oxidation of sugars to sugar acids. Currently, the process of glucose oxidation is carried out biotechnologically with the use of *Aspargilus Niger* and *Gluconobacter suboxydans*. However, it is difficult to separate the product and remove the resulting wastewater. In order to reduce production costs, minimize the amount of waste, and simplify the technologies used, the whole process could be carried out using heterogeneous catalytic systems with the highest possible selectivities. The catalysts should be easily separable from the product, nontoxic, and resistant to deactivation. Monometallic systems have been tested in which the active phase is Pt, Pd or Au. Supported gold catalysts have the best properties, however, the preparation of systems with a high dispersion of gold requires the use of special preparative techniques. Such catalysts, despite their satisfactory activity and selectivity, are still characterized by insufficient stability during the glucose oxidation process. Similarly, the industrial application of supported polymetallic catalysts, e.g., Pd-Bi, Pd-Tl, Pd-Pb, is limited by the elution of metallic components into the reaction solution and thus their low stability. 

In this publication, Pd-Au/SiO_2_ catalysts with different metal loadings were tested for the selective oxidation of glucose. The introduction of gold into the 5% Pd/SiO_2_ system led to an improvement in its catalytic properties, especially its selectivity towards gluconic acid. The activity of the bimetallic catalysts strongly depended on the metal ratio. The highest activity and selectivity towards gluconic acid was obtained for the systems in which the Au/Pd atomic ratio was in the range of 0.05–0.15. We also investigated the long-term stability of the 5% Pd-1% Au/SiO_2_ catalyst. The examined catalyst was successfully used in 10 runs with no observable decrease in activity. The 5% Pd-1% Au/SiO_2_ catalyst showed very high selectivity in each of the 10 runs. The stability data for the Pd-Au/SiO_2_ catalysts presented in this publication are promising, but in order for these catalysts to be used in industrial applications they require even longer stability tests.

The studied Pd-Au/SiO_2_ bimetallic catalysts were also found to be active in the oxidation of glucose produced from cotton waste under the conditions of pressure and temperature-assisted acidic hydrolysis. Gluconic acid was the only reaction product observed in the hydrolysates. Such gluconate-containing products made of cotton waste could be used in surface cleaning fluids or after purification, as intermediates in the cosmetics industry.

Analysis of XRD, ToF-SIMS, SEM-EDS, and TPR data suggested several possible ways in which Au may influence catalytic performance. The addition of gold caused a significant increase in Pd dispersion compared to the monometallic 5% Pd/SiO2 system. At the same time, it seems to have led to the stabilization of small palladium crystallites. The bimetallic Pd-Au/SiO_2_ systems, in which the dispersion of both metallic components was the highest, were characterized by high activity and selectivity towards gluconic acid. The XRD study revealed changes in the crystalline structure of the gold in the bimetallic catalysts, caused by the incorporation of palladium atoms into the crystalline structure of gold. The composition of the formed solid solution depended on the Au/Pd ratio in the bimetallic systems. Analysis of the surfaces of the bimetallic Pd-Au/SiO_2_ catalysts using ToF-SIMS and SEM-EDS techniques, revealed the presence of regions in which both palladium and gold atoms were present. The modification of the electronic and geometric properties of Pd nanocrystals due to their alloying with gold may be an alternative explanation for the better activity and selectivity towards gluconic acid of the Pd-Au/SiO_2_ catalysts. 

To the best knowledge of the authors, who have been following the literature on the catalytic oxidation of aldoses to aldonic acids since the 1990s, there have been no reports of such a process being used commercially, including for the transformation of cotton waste into valuable chemicals. This appears a promising research avenue, with potential industrial applications. 

## Figures and Tables

**Table 1:** Size of palladium and gold crystallites calculated based on XRD measurements in Pd-Au/SiO_2_ bimetallic catalysts, relative to activity, selectivity, and productivity. Reaction conditions: T = 60 °C, m_cat_ = 1g, C_0_ = 1 mol·dm^−3^, pH = 9, V_O2_ = 1 dm^3^·min^−1^.

Catalysts	X * [%]	S * [%]	Productivity of Gluconic Ions	Au/Pd [at/at]	Crystallite Size [nm] ***	
	-	-	[mol·h^−1^·g_Pd_^−1^]		Au	Pd
5% Pd/SiO2	21	51	1.17	0	-	21.3
5% Pd-0.2% Au/SiO2	100	90	4.94	0.02	b.d.l.	5.7
5% Pd-0.5% Au/SiO2	100	90	11.72	0.05	15.1	7.7
5% Pd-1% Au/SiO2	100	99 **	13.57	0.11	8.4	8.2
5% Pd-2% Au/SiO2	49	95 **	5.39	0.22	24.4	5.6
5% Pd-5% Au/SiO2	42	99 **	4.79	0.54	41.3	5.8
5%Au/SiO2	22	70	-	-	43.4	-

* After 2 h of glucose oxidation. ** Gluconic acid was the only product detected by HPLC with Gilson’s prepELS II. *** Crystallite size calculated on the basis of XRD measurements. b.d.l.: Below the detection limit.

**Table 2:** Intensity of the positive ion peaks ^106^Pd and negative ions Au_3_ obtained using the secondary ion mass spectrometry (ToF-SIMS) for bimetallic catalysts Pd-Au/SiO_2_ reduced in H_2_ at 300 °C. Reprinted from ref. [29].

Catalyst	Peak Intensity Pd *·10^6^	Peak Intensity Au_3_ **·10^6^	Relative Intensity Au_3_ **/Pd *
5% Pd-0.2% Au/SiO_2_	1648	42	0.025
5% Pd-0.5% Au/SiO_2_	1727	38	0.022
5% Pd-1% Au/SiO_2_	1961	128	0.065
5% Pd-2% Au/SiO_2_	1984	71	0.035
5% Pd-5% Au/SiO_2_	2062	44	0.021

* ^106^Pd^+^ ion counts/total number of positive ion counts. ** Au_3_^-^ ion counts/total number of negative ion counts.

**Table 3:** ICP-AES analysis of the reaction mixture after catalytic tests. Reaction conditions: T = 60 °C, m_cat_ = 1 g, C_0_ = 1 mol·dm^−3^, pH = 9, V_O2_ = 1 dm^3^·min^−1^.

Catalyst	Pretreatment Steps	Reaction Medium	Pd [ppm] (247.6 nm)	Au [ppm] (242.8 nm)
5% Pd/SiO_2_	H_2_, 2 h, 300 °C	Glucose	0.1148	-
-	H_2_, 2 h, 300 °C	H_2_O	b.d.l.	-
5% Pd-0.5% Au/SiO_2_	H_2_, 2 h, 300 °C	Glucose	0.1056	b.d.l.
	H_2_, 2 h, 300 °C	H_2_O	b.d.l.	b.d.l.
5% Pd-1% Au/SiO_2_	H_2_, 2 h, 300 °C	Glucose	0.0816	b.d.l.
5% Pd-5% Au/SiO_2_	H_2_, 2 h, 300 °C	Glucose	0.0798	b.d.l.
5%Au/SiO_2_	H_2_, 2 h, 300 °C	Glucose	-	0.0257

b.d.l.: Below the detection limit.

**Table 4:** ICP-AES analysis of reaction mixture after stability tests of the 5% Pd-1% Au/SiO_2_ catalyst. Reaction conditions: T = 60 °C, m_cat_ = 1g, C_0_ = 1 mol·dm^-3^, pH = 9, V_O2_ = 1 dm^3^·min^−1^.

Number of Cycle	1	2	3	4	5	6	7	8	9	10
X [%]	100	96	97	90	92	99	96	94	99	94
S [%]	99 *	99 *	99 *	99 *	97 *	96 *	98 *	97 *	96 *	98 *
Pd [ppm] 247.6 nm	0.082	0.145	0.050	0.134	0.049	0.052	0.071	0.102	0.052	0.069
Au [ppm] 242.8 nm	b.d.l.	b.d.l.	b.d.l.	b.d.l.	b.d.l.	b.d.l.	b.d.l.	b.d.l.	b.d.l.	b.d.l.

b.d.l.: Below the detection limit. * Gluconic acid was the only product detected by HPLC with Gilson’s prepELS II detector.

**Table 5:** Acidic hydrolysis of cotton fiber. In each process, 100 g of comminuted cotton yarn (>0.5 cm long) and 2 dm^3^ of the solution of H_2_SO_4_ (concentration 2–10%) were used. The process was carried out between 1 and 4 h in a temperature range of 80–160 °C.

No	Time [h]	C H_2_SO_4_ [%]	Temp. [^o^C]	Dry Mass of Solid Residue [g]	C_Glucose_ HPLC [mol/dm^3^]	Cellulose Crystallinity of the Solid Residue [%]	Product of Cotton Hydrolysis GC-MS Analysis (R.Time)	SEM Images (×500 Magnification) of the Fibers after Hydrolysis
1	2	0	120	92.2	0	91	-	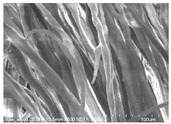
2	1	2	120	86.9	0.008	89	-	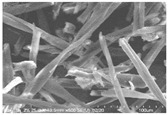
3	2	2	120	81.4	0.010	95	(4.970) Furfural (8.558) 2-Furanocarboxaldehyde, 5-methyl-	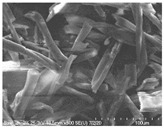
4	4	2	120	82.8	0.015	99	-	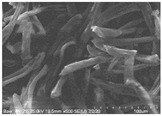
5	2	2	80	91.5	0	90	-	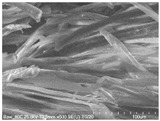
6	2	2	160	42.9	0.020	99	(4.943) Furfural (7.885) 2(5H)-Furanone, 5-methyl- (8.520) 2-Furanocarboxaldehyde, 5-methyl- (12.438) Levulinic acid	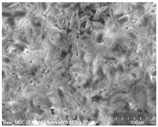
7	2	5	120	81.9	0.018	99	-	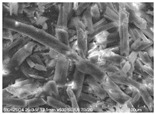
8	2	10	120	84.7	0.018	99	-	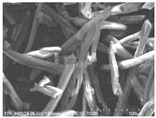

**Table 6:** Conversion degree of glucose and selectivity to gluconate ions in the reaction of glucose from hydrolysates of cotton in the presence of 5% Pd-1% Au/SiO_2_ catalyst. Reaction conditions: T = 60 °C, pH = 9, t = 2 h, V_O2_ = 1 dm^3^·min^−1^, V_GLU_ = 0.25 dm^3^.

Sample	C_0 GLU_ [mol·dm^−3^]	m_cat_ [g]	X [%]	S_GLC_ [%]
1	0.02	0.02	100	100
2	0.04	0.04	100	100
3	0.08	0.08	100	100

## Data Availability

The data presented in this study are available on request from the corresponding author.
